# A Vision for VenomsBase: An Integrated Knowledgebase for the Study of Venoms and Their Applications

**DOI:** 10.1093/iob/obaf026

**Published:** 2025-06-27

**Authors:** T A Castoe, M Daly, F Jungo, K N Kirchhoff, I Koludarov, S Mackessy, J Macrander, S Mehr, M V Modica, E E Sanchez, G Zancolli, M Holford

**Affiliations:** Department of Biology, University of Texas at Arlington, Arlington, TX 76019, USA; Department of Evolution, Ecology, and Organismal Biology, The Ohio State University, Columbus, OH 43210, USA; SIB Swiss Institute of Bioinformatics, Swiss-Prot Group, 1211 Geneva , Switzerland; Department of Chemistry, Hunter College, City University of New York, New York, NY 10065, USA; TUM (Technical University of Munich), School of Computation, Information and Technology (CIT), Faculty of Informatics, Chair of Bioinformatics & Computational Biology - i12, Boltzmannstr. 3, 85748 Garching/Munich, Germany; Department of Biological Sciences, University of Northern Colorado, Greeley, CO 80639, USA; Biology Department, Florida Southern College, Lakeland, FL 33801, USA; Department of Chemistry, Hunter College, City University of New York, New York, NY 10065, USA; Stazione Zoologica Anton Dohrn, Department of Biology and Evolution of Marine Organisms, Roma 80121, Italy; Department of Chemistry, National Natural Toxins Research Center, Kingsville, TX 78363, USA; Department of Ecology and Evolution, University of Lausanne, Lausanne 1015, Switzerland; Department of Chemistry, Hunter College, City University of New York, New York, NY 10065, USA; Department of Invertebrate Zoology, The American Museum of Natural History, New York, NY 10024, USA; Programs in Biology, Biochemistry, and Chemistry at CUNY Graduate Center, New York, NY 10016, USA

## Abstract

Venoms are complex bioactive mixtures that have independently evolved across diverse animal lineages, including snails, insects, sea anemones, spiders, scorpions, and snakes. Despite the growing interest in venom research, data is fragmented across disparate databases which lack standardization and interoperability. A vision for the proposed VenomsBase platform presented here seeks to address these challenges by using the best practices approach in creating a centralized, open-access platform adhering to FAIR principles (Findable, Accessible, Interoperable, and Reproducible). VenomsBase will unify venom datasets, standardize terminology, and enable comparative analyses across species, facilitating novel toxin discovery and functional annotation. Key features of VenomsBase include user-friendly data submission modules with built-in validation, advanced cross-species analysis tools, and integration of multidisciplinary datasets spanning genomics, transcriptomics, proteomics, functional assays, and ecological metadata. A modular, cloud-based design will ensure scalability, while heuristic scoring systems will guide users toward high-confidence data entries. To promote accessibility, the envisioned VenomsBase will provide tutorials, regular training sessions, case studies, and feedback loops, supporting researchers at all levels. By harmonizing venom research and addressing the limitations of outdated or nonstandardized methods, VenomsBase aims to revolutionize the field, while being continuously improved and refined by venom experts. This initiative will unlock venoms’ potential to make groundbreaking discoveries, address global health challenges, and foster collaboration and innovation across the scientific community.

## Italian

I veleni sono complesse misture bioattive che si sono evolute indipendentemente in diversi linee evolutive di animali, fra i quali lumache marine, insetti, anemoni di mare, ragni, scorpioni e serpenti. Nonostante il crescente interesse nei confronti della ricerca sul veleno, i dati ad oggi accumulati sono frammentati in diversi database privi di standardizzazione e interoperabilità. In questo lavoro viene presentata la visione che ispira la proposta della piattaforma VenomsBase, formulata per superare queste limitazioni basandosi sulle best practices disponibili. VenomsBase sarà una piattaforma centralizzata e open-access, in linea con i principi FAIR (Findable, Accessible, Interoperable, and Reproducible). VenomsBase unificherà i dataset sul veleno e standardizzerà la terminologia, permettendo analisi comparative fra specie diverse e facilitando la scoperta di nuove tossine e la loro annotazione funzionale. Le caratteristiche principali di VenomsBase includono la sottomissione di dati tramite moduli user-friendly con validazione incorporata, la disponibilità di strumenti avanzati di analisi interspecifica, e l'integrazione di dataset multidisciplinari comprendenti genomica, trascrittomica, proteomica, saggi funzionali e metadati ecologici. Un design modulare, basato su cloud, ne assicurerà la scalabilità, mentre un sistema euristico a punteggio guiderà gli utenti nell'inserimento di dati robusti e affidabili. L'accessibilità di VenomsBase verrà promossa tramite l'offerta di tutorial, sessioni periodiche di training, presentazione di casi studio, e interazione continua con gli utenti, supportando i ricercatori a tutti i livelli. Armonizzando la ricerca sui veleni e superando le limitazioni dovute a metodi arretrati o non standardizzati, con miglioramenti continui da parte della comunità di esperti di riferimento, VenomsBase si propone di rivoluzionare lo studio dei veleni animali.

Questa iniziativa faciliterà la completa realizzazione del potenziale dei veleni animali nel condurre a scoperte pionieristiche, affrontare sfide globali per la salute umana, e promuovere collaborazione e innovazione nella comunità scientifica.

## German

Tiergifte sind komplexe bioaktive Gemische, die sich unabhängig voneinander in zahlreichen Tierlinien entwickelt haben, darunter Schnecken, Insekten, Seeanemonen, Spinnen, Skorpione und Schlangen. Trotz des wachsenden Interesses an der Tiergiftforschung sind die Daten auf zahlreiche Datenbanken verteilt, die nicht standardisierte sind oder untereinander operieren. Die hier vorgestellte Vision einer VenomsBase-Plattform zielt darauf ab, diese Herausforderungen zu lösen, indem sie eine zentralisierte, frei zugängliche Plattform auf die bestmöglichste Art und nach den FAIR-Prinzipien (Findable = Auffindbar, Accessible = Zugänglich, Interoperable = Interoperabel, Reproducible = Reproduzierbar) aufbaut. VenomsBase wird dabei Datensätze zahlreicher Tiergifte vereinheitlichen, die Terminologie standardisieren und vergleichende Analysen zwischen den Gifttierarten ermöglichen. Dies wird die Entdeckung neuer Toxine und deren funktionelle Annotation erleichtern. Zu den Hauptmerkmalen von VenomsBase gehören benutzerfreundliche Dateneingabemodule inklusive Validierung, fortschrittliche Analysewerkzeuge für den Vergleich zwischen verschiedenen Arten sowie die Integrierung multidisziplinärer Datensätze aus den Bereichen Genomik, Transkriptomik, Proteomik, funktionelle Assays und ökologische Metadaten. Ein modularer, cloudbasierter Aufbau gewährleistet Skalierbarkeit, während heuristische Bewertungssysteme die Nutzer zu besonders zuverlässigen Dateneinträgen führen. Um die Zugänglichkeit weiter zu fördern, wird VenomsBase Lehrmethoden, regelmäßige Schulungen, Fallstudien und Feedback-Schleifen anbieten und Forschende aller Erfahrungsstufen unterstützen. Durch Datenharmonisierung in der Tiergiftforschung sowie durch neue Strategien im Umgang mit den Limitierungen von veralteten oder nicht standardisierter Methoden möchte VenomsBase das Forschungsfeld revolutionieren und sich dabei kontinuierlich unter Mitwirkung von Giftexpert: innen weiterentwickeln. Diese Initiative wird das Potenzial von Tiergiften zugänglicher machen und somit bahnbrechende Entdeckungen ermöglichen, globale Gesundheitsherausforderungen adressieren und Zusammenarbeit sowie Innovation in der wissenschaftlichen Gemeinschaft vorantreiben.

## French

Les venins sont des cocktails bioactifs complexes qui ont évolué indépendamment au sein de diverses lignées animales, incluant les cônes, les insectes, les anémones de mer, les araignées, les scorpions et les serpents. Malgré l'intérêt croissant de la recherche sur les venins, les données sont dispersées au sein de bases de données disparates avec une faible standardisation et peu d'interopérabilité. L'idée de la plateforme VenomsBase, présentée ici, cherche à surmonter ces difficultés en utilisant la meilleure approche pratique en créant une plateforme libre d'accès centralisée adhérant aux principes FAIR (Facile à trouver, Accessible, Interopérable et Reproductible). VenomsBase compilera des ensembles de données, standardisera la nomenclature et permettra des analyses comparatives entre espèces, facilitera la découverte et l'annotation fonctionnelle de nouvelles toxines. Les points clés de VenomsBase incluent une soumission de modules simple d'utilisation avec une validation intégrée, des outils d'analyse inter-espèces avancés, ainsi qu'une intégration de jeux de données pluridisciplinaires couvrant la génomique, la transcriptomique, la protéomique, les essais fonctionnels et les métadonnées écologiques. Une architecture modulaire, basée sur le *cloud*, garantira l'évolutivité, tandis que des systèmes de *scoring* heuristiques guideront les utilisateurs vers des saisies de données à haute fiabilité. Pour promouvoir l'accessibilité, la plateforme VenomsBase envisagée fournira des tutoriels, des sessions de formation régulières, des études de cas, et un suivi, afin d'accompagner les chercheurs à tous les niveaux. En harmonisant la recherche sur les venins et en s'affranchissant des limites de méthodes obsolètes ou non-standardisées, VenomsBase a pour objectif de révolutionner le secteur, tout en étant continuellement améliorée et perfectionnée par des experts en venins. Cette initiative libérera le potentiel des venins pour faire des découvertes révolutionnaires, relever des défis sanitaires mondiaux et favoriser la collaboration et l'innovation au sein de la communauté scientifique.

## Portuguese

Os venenos são misturas bioativas complexas que evoluíram independentemente em diversas linhagens animais, incluindo caracóis, insetos, anêmonas-do-mar, aranhas, escorpiões e serpentes. Apesar do crescente interesse na pesquisa de venenos, os dados disponíveis encontram-se fragmentados em *datasets* díspares, muitas vezes carentes de padronização e interoperabilidade. A proposta da plataforma VenomsBase, apresentada neste trabalho, visa enfrentar esses desafios por meio da adoção de boas práticas no desenvolvimento de uma plataforma centralizada, de acesso aberto, fundamentada nos princípios FAIR: Encontrável, Acessível, Interoperável e Reutilizável (do inglês, Findable, Accessible, Interoperable, and Reusable). A VenomsBase unificará os conjuntos de dados de venenos, padronizará a terminologia e permitirá análises comparativas entre espécies, facilitando a pesquisa de novas toxinas e a anotação genômica. As principais funcionalidades da plataforma incluem módulos de entrada de dados de fácil uso com validação integrada, ferramentas avançadas para análise interespécies e integração de dados multidisciplinares que abrangem genômica, transcriptômica, proteômica, ensaios funcionais e metadados ecológicos. Um design modular baseado em nuvem eletrônica garantirá a escalabilidade, enquanto os sistemas de pontuação heurística auxiliarão os usuários na identificação de dados com alto grau de confiabilidade. Para promover a acessibilidade, a VenomsBase oferecerá tutoriais, sessões regulares de treinamento, estudos de caso e ciclos de feedback, dando suporte a pesquisadores de todos os níveis. Ao harmonizar a pesquisa dos venenos e abordar as limitações de métodos desatualizados ou não padronizados, esta iniciativa visa revolucionar o campo, sendo continuamente aprimorada por especialistas da área. A VenomsBase pretende desbloquear o potencial dos venenos para descobertas científicas inovadoras, o enfrentamento de desafios globais em saúde e o estímulo à colaboração e à inovação na comunidade científica.

## Spanish

Los venenos son mezclas bioactivas complejas que han evolucionado de forma independiente en diversos linajes evolutivos de animales como caracoles, insectos, anémonas de mar, arañas, escorpiones y serpientes. A pesar del creciente interés por la investigación en venenos animales, los datos están fragmentados en distintas bases de datos dispares que carecen de estandarización e interoperabilidad. La propuesta de la plataforma *VenomsBase*, aquí presentada, busca abordar estos desafíos mediante la adopción de buenas prácticas con el objetivo de crear una plataforma centralizada y de libre acceso que se adhiera a los principios FAIR: Localizable, Accesible, Interoperable y Reutilizable (del inglés, Findable, Accessible, Interoperable, and Reusable). *VenomsBase* armonizará los conjuntos de datos provenientes de venenos, estandarizará la terminología y permitirá realizar análisis comparativos entre especies, facilitando el descubrimiento de nuevas toxinas y la anotación genómica. Entre las características principales de *VenomsBase* se incluyen módulos fáciles de usar para el ingreso de datos con validación incorporada, herramientas avanzadas de análisis entre especies e integración de datos multidisciplinares, los cuales abarcan la genómica, la transcriptómica, la proteómica, los ensayos funcionales y los metadatos ecológicos. Su arquitectura modular basada en la nube garantizará la escalabilidad, mientras que los sistemas heurísticos de puntuación guiarán a los usuarios hacia los datos de alta confianza. Para fomentar la accesibilidad, *VenomsBase* ofrecerá tutoriales, sesiones de capacitación periódicas, estudios de caso y canales de retroalimentación, brindando apoyo a investigadores de todos los niveles. Al armonizar la investigación sobre venenos y abordar las limitaciones de métodos obsoletos o no estandarizados, esta iniciativa busca revolucionar el campo, al tiempo que constantemente será mejorada y refinada por expertos en venenos. *VenomsBase* aprovechará el potencial de los venenos para realizar descubrimientos innovadores, enfrentar desafíos globales en salud y fomentar la colaboración y la innovación dentro de la comunidad científica.

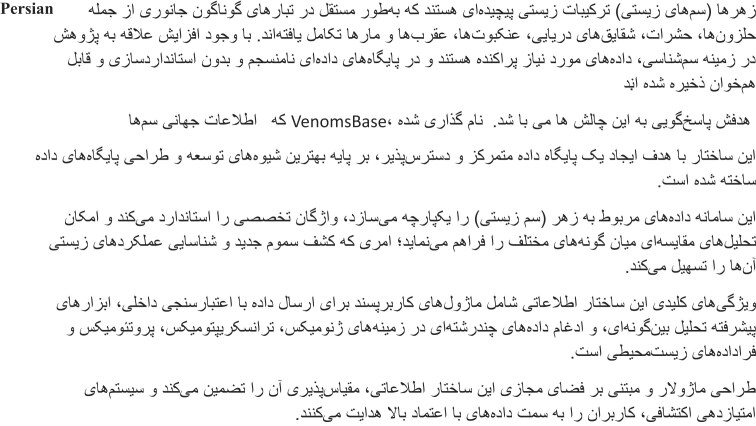

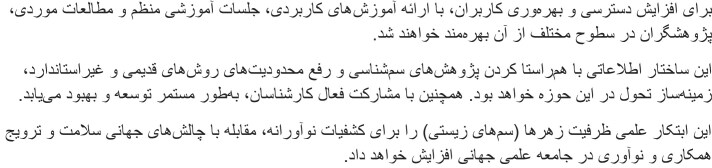

## Introduction

**Fig. 1.**
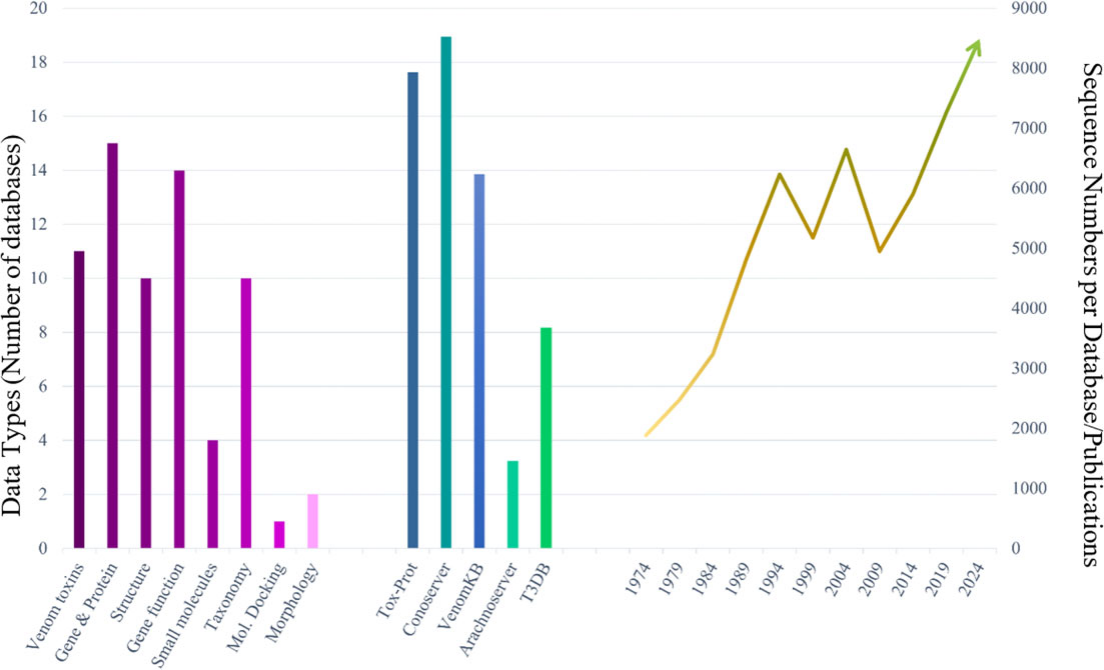
Venom-related data currently stored across various databases in a highly decentralized manner organized by data types (left; from Zancolli et al. 2024), encapsulating sequence data as either being taxon specific or broad taxonomic groups (center) with an increasing number of publications in NCBIPubMed under the keyword “venom” (right).

Venoms are complex bioactive cocktails of proteins, peptides, and other compounds that have evolved independently across diverse animal lineages, including snails, insects, sea anemones, spiders, scorpions, and snakes (Schendel et al. 2019). The venom of a single species may contain >100 biologically active proteins and peptides, encoded by >20 gene families (Casewell et al. 2013). The broad range of animals that produce venom, along with the physiological mechanisms underlying venom production, serve as valuable models for studying the evolution, genetics, and physiology of how new genes, gene regulatory networks, protein function, and phenotypes arise in nature (Vonk et al. 2013; Zancolli and Casewell 2020; Barua and Mikheyev 2021; Perry et al. 2022; Schield et al. 2022). The biochemical effects of venoms, their biotechnological applications and subsequent impact beyond basic biology, intersect with a variety of fields, including pharmacology, neuroscience, and immunology. Many venom components have been harnessed as valuable resources for therapeutic and biomedical applications, leading to breakthroughs in pain management, cardiovascular treatments, and metabolic therapies (King 2015; Holford et al. 2018; Muttenthaler et al. 2021). Venom's applications in biotechnology continue to expand, with venom-derived peptides inspiring the development of drugs like Ozempic for metabolic disorder and obesity treatment (Christou et al. 2019). Conversely, venoms also pose significant public health challenges, particularly in tropical and subtropical regions where envenomation from snakes, scorpions, and other venomous organisms remains a major cause of morbidity and mortality (Kasturiratne et al. 2008; Patikorn et al. 2022; Puzari et al. 2025), with more than 125,000 dying annually from snakebites alone (Afroz et al. 2024). Research on venoms therefore transcends numerous diverse disciplines. Despite the scientific and commercial promise of venom research (Fig. 1), major obstacles limit its application and potential, stemming from critical gaps in data infrastructure centralization, standardization, and organization (Frisvold et al. 2021).

The growth in venom research has resulted in venom-related data in disparate databases, such as UniProtKB/Tox-Prot (Jungo et al. 2012), ArachnoServer (Pineda et al. 2018), and Conoserver (Kaas et al. 2011), which vary greatly in size, scope, and curation standards (Fig. 1, Jungo et al. 2010). Currently, venom peptides, proteins, and other bioactive components are analyzed and stored from multiple research disciplines, generating a wide range of databases that host raw, quality-controlled, or specialized datasets. However, the diversity of fragmented datasets, with different standards, and limited cross-disciplinary collaboration have constrained venom research (e.g., Kuzmenkov et al. 2016; Batko and Ślęzak 2022; Dresler et al. 2024). To date, three main databases store venom protein and peptide sequences. UniProtKB/Tox-Prot contains 8055 curated entries (release 2025_01) spanning a wide variety of venomous animals and principally covers toxic compounds with functional characterization. In comparison, ConoServer is dedicated to just cone snail venom compounds, and holds a total of 8523 entries, of which, 3058 correspond to wild-type protein entries (as of February 2025). This distinction allows a more accurate comparison with other databases and highlights the differing standards across venom databases. For its part, ArachnoServer includes 1458 curated spider entries (as of February 2025), representing a slight decrease compared to the 1569 spider entries listed in Tox-Prot. Furthermore, to compare the number of entries in ConoServer and ToxProt, we would have to consider the wild-type entries in Conoserver, that is, 3058 entries. The difference in the number of entries between Tox-Prot and ConoServer stems from their distinct annotation standards. ToxProt displays one entry per gene, incorporating studies on synthetic variant/mutant sequences within these entries. In contrast, ConoServer presents two entries for the same protein (one for the mature peptide and another for the precursor) and provides a new entry for each synthetic variant/mutant sequence. This discrepancy may be attributed to the update frequencies and inclusion criteria between the databases. In addition to the three main databases, the web portal VenomZone offers detailed information on venoms as well as their molecular targets. Users can browse data by taxonomy, activity, or venom protein families, with each page linking to related proteins in Tox-Prot, organized by species or protein family (Zancolli et al. 2024). Fragmented, incomplete, and disconnected taxon-specific datasets with different standards currently limit venom research and its broader potential applications and impact.

Creating a centralized, standardized, and expertly curated platform for venom research is essential to unify existing and novel data resources, mitigate biases, identify shared patterns, and establish consistent gene and/or protein family classifications (Zancolli et al. 2024). Such venom data integration would facilitate a comprehensive understanding of venom diversity and function, and unlock significant bioeconomic opportunities in the pharmaceutical, biotech, agricultural, and cosmetic sectors. Another issue is defining what counts as a venom protein or compound. This is very much an open question and part of an ongoing sustainability issue without a foundational framework to build onto. Here we propose an initiative to address these issues that we refer to as VenomsBase. This initiative responds to calls to establish a transformative infrastructure platform that removes both the technological (intrinsic) and the organizational (extrinsic) barriers that are preventing venom-focused research in achieving its full potential. The envisioned VenomsBase will support the advancement of venom research by providing a process to explore different approaches, discuss opportunities and challenges, and ultimately enact some transparent approaches to defining venom (or venom proteins). In constructing VenomsBase we are fully considering the openness of these questions as assets and a rationale for building a community-driven resource.

## Barriers to progress in venoms research

Understanding the barriers to modern venom research requires examining three key factors: the historical legacy of venom discovery, the complex genetics underlying venom production, and how the negative interaction between these elements has created confusion, scientific siloing, and inconsistencies in the field. For decades, most of the work in the venom field has been at the translational level, surveying the diversity and biological activity of whole venom cocktails or individual venom peptides and proteins. The original emphasis was on a few lineages and approaches, being more descriptive rather than comparative. Consequently, naming conventions for venom components largely represent their observed biological activity (e.g., mastoparan), are not connected across lineages, and do not reflect the genes that encode these venoms or their shared (or distinct) origins (Oliveira et al. 2012; Hargreaves and Mulley 2014). Progress in understanding the genomic basis of venoms has developed only recently, as previously hindered by the unique challenges of how venom genes are encoded in animal genomes (Zelanis and Keiji Tashima 2014; Sunagar et al. 2016; von Reumont et al. 2022a). Venom proteins tend to be encoded by complex multicopy gene arrays, which vary in coding sequence and copy number substantially between and even within species (Wong and Belov 2012; Martinson et al. 2017; Gopalan et al. 2022; Smith et al. 2023). Post-transcriptional and -translational modification (PTMs) further complicate the relationship between genes, proteins, and their function for venoms (Ogawa et al. 2019; Ye et al. 2023). Together, these factors significantly complicate, and thus limit our understanding of basic information about the number and diversity of venoms, post curation, encoded in the genomes of otherwise well-studied species. Accordingly, a fundamental barrier to progress is the disconnection between nearly 50 years of studies on venom-derived peptides/proteins and their biological properties, their fundamental relationships with one another, and with the genetic and genomic elements that encode and regulate these proteins.

Another major barrier is that the existing curated and publicly available resources include broad, but nonspecific databases like NCBI and UniProtKB (The UniProt Consortium 2025) and smaller specific databases like ArachnoServer (Pineda et al. 2018), and ConoServer (Kaas et al. 2011) (Fig. 1). While these databases have been instrumental in advancing venom research, they remain limited in scope, focusing on specific types of data or lineages, and often having unique notation schemes for post-translational modifications or nonstandard amino acids. Critically, they are not designed to address broader cross-disciplinary challenges across diverse venomous lineages, such as the accurate and standardized curation and annotation of venom genes from genome, transcriptome, and protein datasets. The current databases are also not designed to support comparative analyses and are limited in terms of data annotation for both function and taxa. Furthermore, these resources are not scalable: in some cases, data formats and infrastructure render them largely incompatible or restricted in their interoperability, and in other cases the data are presented partially with regard to searchability or summarization (European Commission: Directorate-General for Research and Innovation 2018; Holmes 2018; Sima et al. 2019; Di Muri et al. 2024). Finally, for most of the existing venom databases, the user interface is challenging to navigate, often requiring specialized tools or software. This makes user accessibility a substantial barrier, presenting a steep learning curve for many early career scientists or those without bioinformatics expertise. Taken together, the shortcomings of existing resources indicate that no single researcher or research group possesses the information or technical capability to generate or interpret the scope of data associated with venom research. Consequently, venom research's complexity and interdisciplinary nature emphasize the need for a multimodal integrated database like VenomsBase to improve data accessibility and collaboration (Dresler et al. 2024).

Indeed, a decade of community-building among researchers studying venoms has identified the need for data standardization and integration. Convening events, such as the biennial Gordon Research Conference (Venom Evolution, Function and Biomedical Applications) and the annual World Congress of the International Society of Toxinology, often include community planning and prioritizing sessions that routinely highlight how existing infrastructure fails to adequately support the large-scale, multidisciplinary data sharing and analysis necessary to accelerate venom research outcomes. This key issue was also thoroughly discussed within the COST Action European Venom Network (EUVEN), an EU-funded community-building initiative that gathered researchers in the field in the past 4 years (Zancolli et al. 2024). These conversations echo nearly all recent review manuscripts in the field of venom research, which call for approaches that facilitate the cross-interrogation of diverse data, across lineages and datatypes, to explore shared attributes of venoms, model the structure of venom molecules, and search for bioactive compounds in community-accessible formats (von Reumont et al. 2022b; Prentis et al. 2018; Schendel et al. 2019; Arbuckle 2020; DiFrisco and Jaeger 2020; Kini 2020; Smallwood and Clark 2021; Avella et al. 2022; de Oliveira et al. 2023; Calvete et al. 2024). VenomsBase was conceptualized as a response to this community need by providing leading-edge infrastructure that enables comparative investigation of venoms and their associated genomic regions across species and facilitates curated multispecies genotype-to-phenotype datasets.

## Proposed solution: creation of an integrated knowledgebase—VenomsBase

**Fig. 2.**
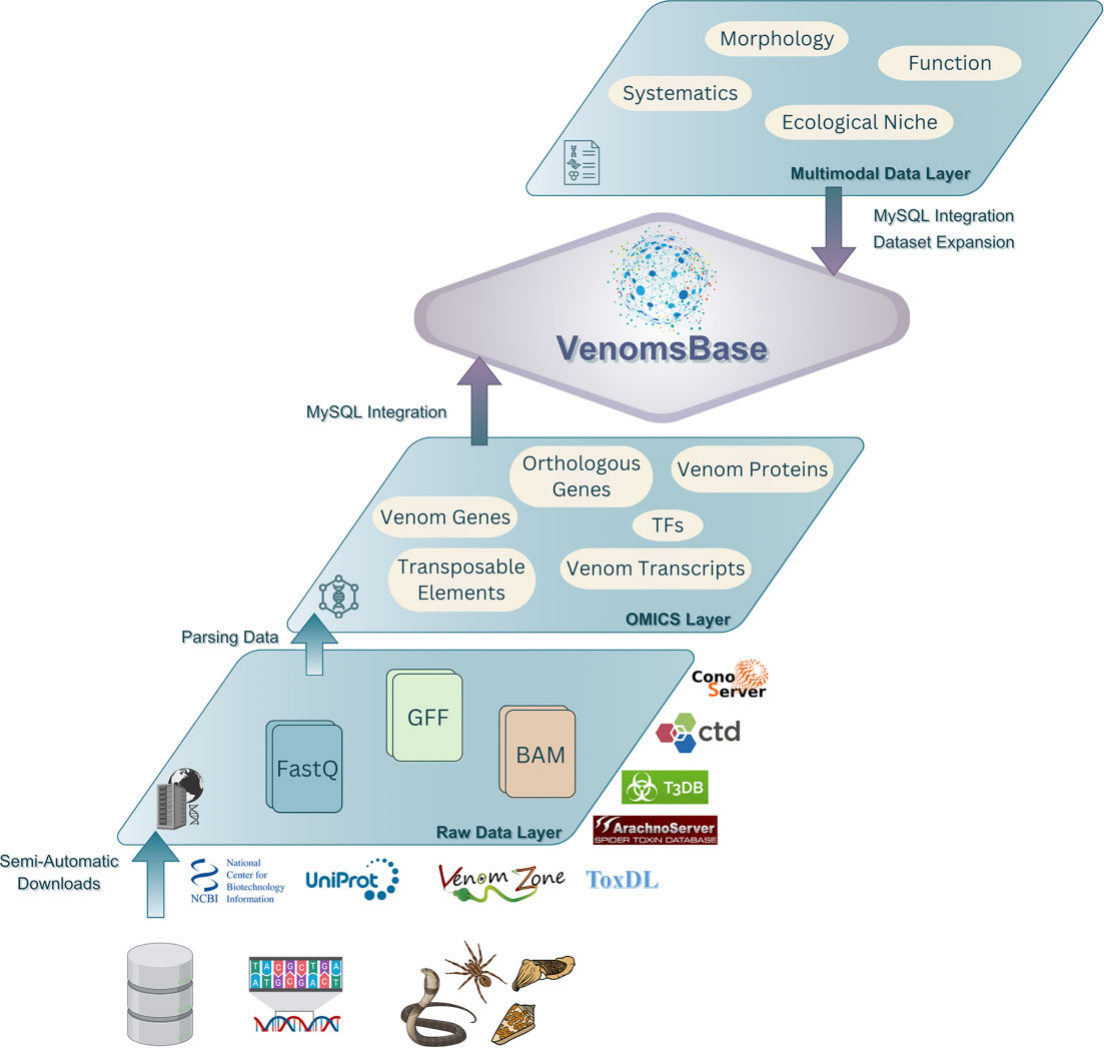
Schematic overview of VenomsBase's modular structure for data processing and integration. The Raw Data Layer gathers datasets via semi-automatic downloads, which are processed in the Parsing Data Step for high-quality integration. The OMICS Layer curates venom-related omics data for comprehensive curation and standardization. The Dataset Expansion Step continuously incorporates new datasets into the Multimodal Data Layer, enabling cross-disciplinary analyses. This modular framework enhances scalability, interoperability, and data diversity in venom research.

The VenomsBase initiative described here would establish a global venom data integration platform to resolve critical challenges in venom research, such as data fragmentation, inconsistent characterization between venom proteins and the genes that encode venoms, and inconsistent terminology for venom protein families (Oliveira et al. 2012; Hargreaves and Mulley 2014). The transformative impact of VenomsBase's infrastructure is based on its overarching theme of integrating standardized knowledge across biological scales to associate venom proteins to the genes and genomes that encode them, and from the physiological secretory systems and whole organisms that produce them, spanning the animal tree of life (Fig. 2). Given the high level of multidisciplinary venom research, data consistency and robust quality are inherently challenging and require coordinated curation and participation at a community scale. VenomsBase is intended to fill this gap by integrating genome-to-phenome datatypes to centralize, standardize, and make accessible naming conventions for venom proteins, gene families, and isoforms, while further developing standardized reference sets of venom gene/transcript models coupled to the proteins these genes produce. Our vision for the VenomsBase initiative would enable robust data curation and refinement over time and provide the community with a highly vetted standard foundation for studying, comparing, and applying research on venoms and venomous animals. We argue that such an ambitious initiative could be accomplished through three distinct phases of prioritized development: (1) Establishment of a standardized and scalable platform for integration of diverse platinum-quality venomous reference species; (2) Development of cross-species search and analysis tools and expansion of taxa representation; and (3) Integration of multidisciplinary data types through collaborator engagement and feedback.

Through these prioritized development strategies, VenomsBase would establish valuable infrastructure to the research community by lowering barriers to entry in terms of expertise and access to analytical tools, thereby accelerating collaboration, discovery, and applications of venom-related research. This will be done via collaborative message boards and GitHub repositories to establish a centralized communication platform to coordinate discussions, share updates, and solicit feedback from community members. The envisioned VenomsBase platform would address gaps of siloed and species-specific datasets by developing a central infrastructure for submitting, standardizing, and analyzing multispecies venom-related data across labs worldwide that is open-access and Findable, Accessible, Interoperable, and Reproducible (FAIR) (Wilkinson et al. 2016, 2024). VenomsBase will draw on successful aspects of VenomKB v2.0 as described by Romano and Tatonetti (2015) and of established databases like EchinoBase (Arshinoff et al. 2022), ArachnoServer (Pineda et al. 2018), and ConoServer (Kaas et al. 2011) to create a centralized, structured resource that consolidates venom taxonomy, bioactivities, sequences, and structural data (Fig. 2). VenomsBase will incorporate elements of other successful annotation pipelines, like Funannotate (Palmer and Stajich 2020) or similar to standardized annotation workflows, and incorporate Ensembl-guided gene model predictions (Dyer et al. 2025) that will be functional across species in order to track annotations and expand with VenomsBase. Via an ontology-driven structure, VenomsBase would ensure consistency in data classification while integrating diverse data types, such as genomic sequences, gene/transcript annotations, proteomic profiles, functional assays, taxon images, and pharmacological interactions, thus establishing a comprehensive resource for biological and chemical diversity, computational toxinology, and bioeconomic development. VenomsBase would prioritize user accessibility and data quality, adopting features like heuristic annotation scoring to guide users toward the most robust entries and programmatic access through a REST API, as exemplified by VenomKB v2.0 (Romano and Tatonetti 2015). By incorporating features from other databases, such as visualization tools for post-translational modifications and curated protein structural data links, such as UniProtKB/Tox-Prot (Jungo et al. 2012), VenomsBase will provide a streamlined interface for venom research, allowing users to explore data by specific compounds, molecular targets, species, and translational applications (Jungo et al. 2010). Taken together, VenomsBase will integrate with the community to establish new standards and data practices, using tractable and informative methods, and test new processes to integrate data across broad scales.

By unifying knowledge of the genes and regulatory networks that produce venoms with the structure and function of venom proteins and peptides, VenomsBase aims to create a comprehensive resource that links venom composition to its biological targets and physiological effects (Fig. 2). Our multi-institutional team is leading the initial curation effort to secure infrastructure grants and establish a long-term consortium for sustainability and maintenance. This effort is modeled on successful large-scale data repositories such as the World Register of Marine Species (WoRMS), which relies on a distributed network of backend and scientific experts with clearly defined roles (Ahyong et al. 2025), including taxonomists, web developers, and various programmers. VenomsBase will require not only scientific curators but also dedicated developers and database administrators. Developers will maintain platform infrastructure, while database administrators will manage data ingestion from generalist repositories (e.g., NCBI, UniProt), validate submission formats, and coordinate public data releases. Automated workflows will follow lineage-specific filtering rules (e.g., taxonomic identifiers, venom-related GO terms, expression in venom glands) to ensure relevant data capture. This tiered structure mirrors existing systems such as UniProt, which handles diverse types of submitted sequences, including rejections and redirection to repositories like GenBank or PRIDE. This integration is expected to lead to transformative research across biological scales, from evolutionary to organismal–encompassing genomes, gene regulatory networks, transcripts, proteins, cell morphology, and biological activity and applications of venoms for selected number of species. As VenomsBase grows, we plan to formalize these roles through institutional partnerships and funded positions. From a global health perspective, VenomsBase would assist with the discovery and development of treatments for envenomations, which remain a significant challenge around the globe in tropical and subtropical regions.

## VenomsBase uses a standardized and scalable approach

Developing a standardized and scalable approach is critical to ensuring VenomsBase's success and adoption by the research community and its utility for ecologists, pharmacologists, and others working with any venomous species across the Tree of Life. This will involve designing workflows and pipelines based on multiple “platinum-quality” species as proof-of-concept and as a means of developing gold-standard practices. Platinum-quality species are those for which there are diverse, high-quality data of multiple types—from genomic, transcriptomic, proteomic resources to anatomical, ecological, and toxinological observations. Gold standard practices include rigorous data validation steps to ensure data integrity and quality, a robust system for annotating and curating datasets, and clear protocols for integrating new data types as they become available.

The key elements of the envisioned VenomsBase platform include:

1. **Data collection and submission modules**: A user-friendly interface to allow researchers to upload datasets to NCBI with built-in validation steps to ensure correct formatting and metadata inclusion. This feature will maintain consistency and quality across datasets. The design should incorporate public datasets as a gateway to support existing practices to curate data in the National Center for Biotechnology Information (NCBI) and the Sequence Read Archive (SRA).
2. **Standardization protocols**: Reproducibility, shareability, and comparability of results are critical in overcoming the challenges of interdisciplinary research, despite increasing data availability and computational power. The infrastructure should employ a terminology editor application to ensure uniform naming conventions and nomenclature. This approach will minimize inconsistencies and improve data interoperability (Lehne et al. 2019; Politano et al. 2019; Rahrooh et al. 2024; Zancolli et al. 2024).
3. **Cross-species tools**: Advanced search and analysis tools will enable researchers to perform comparative analyses across species, leveraging insights from shared genetic and phenotypic traits. The platform should support taxonomically diverse lineages and predicted homologous or analogous molecular models through tools like MetamORF (Choteau et al. 2021), Orthofinder (Emms and Kelly 2019), and methods targeting short open reading frames (Coelho et al. 2024) for annotating novel toxin discovery (Nachtigall et al. 2021) across multiple venomous lineages (Nachtigall et al. 2024). In doing so, the platform would incorporate expert manual curation. As the platform grows, experts across relevant fields will guide manual curation through a tiered model, with a core team for quality control, invited experts for lineage-specific review, and community volunteers who contribute annotations or feedback through the open platforms.
4. **Integration of multidisciplinary data types**: The infrastructure should incorporate diverse datasets, including genomic sequences, transcriptomic data, proteomic profiles, species distributions, and ecological metadata, to create a resource spanning biological scales. Although such a multimodal dataset does not yet exist for venoms, successful frameworks such as the Global Biodiversity Information Facility (GBIF.org. 2025) or platinum-quality species-specific repositories (e.g., Berardini et al. 2015; Sternberg et al. 2024; Öztürk-Çolak et al. 2024) can serve as models for developing this infrastructure.
5. **Scalability and modular design**: The platform should employ modular, cloud-based architecture to allow for growth and adaptation to emerging research needs. It should also integrate heuristic annotation scoring systems to guide users toward high-confidence data entries. Similar heuristic processes have been successfully implemented in genomic next-generation sequencing pipelines (e.g., McLaren et al. 2016; Afgan et al. 2018), particularly in open-source and modular applications (Treangen et al. 2013; Breitwieser et al. 2022).
6. **Training and outreach**: To ensure accessibility and usability for researchers at all levels, the platform should provide regular webinars, training sessions, and user feedback loops. Case studies and tutorials can demonstrate the platform's ability to streamline venom NGS workflows while promoting accessibility through educational resources (Lowman et al. 2009; Shaffer et al. 2010; Brazas and Ouellette 2013; i5K Consortium 2013; Clark et al. 2016; Wilkinson et al. 2016; Attwood et al. 2019; Auwera and O'Connor 2020).

By adopting these elements, VenomsBase would establish a reliable and adaptable foundation to the evolving needs of the venoms and ecological research communities. Incorporating platinum-quality species and agreed-upon protocols will provide a benchmark for data quality, setting a new precedent for collaborative research and discovery.

To democratize access to its resources, the design of databases underlying VenomsBase would prioritize FAIR principles (Wilkinson et al. 2016, 2024). Accessibility is particularly critical for researchers in biodiversity-rich regions like Southeast Asia, Africa, and Latin America, where researchers often face barriers to accessing advanced bioinformatics tools (Geneviève et al. 2018) and where envenomation poses the most significant public health risk. Developing accessible online educational programs tailored to venom research would be another crucial step in bridging the resource gap. Establishing international collaborations between researchers in biodiversity-rich but resource-limited regions and institutions in well-funded countries would enable comprehensive comparative studies, linking venom composition and actions to ecological variables such as climate, prey availability, and habitat type. Additionally, regional connectivity would facilitate the identification of overlooked venomous species, ensuring that antivenom research is informed by an accurate understanding of local venom and species diversity.

## Development of an infrastructure that keeps pace with innovation across fields

To ensure success, VenomsBase future data infrastructure and pipelines must actively facilitate interdisciplinary collaboration, enabling contributions from scientists both within and on the periphery of venom research. This inclusiveness will make the platform more robust and nimble to anticipate new technologies, data types, and analyses. It also expands the scope of questions that can be addressed and reinforces and extends the quality of the protocols through broad and rigorous cross-validation. This approach is also critical for integrating diverse perspectives and expanding the boundaries of the field. Achieving the degree of inclusive flexibility needed hinges on the standardization of data formats, ensuring that datasets are accessible, interpretable, and compatible for researchers across disciplines, including bioinformatics, ecology, physiology, evolutionary biology, and many others (Kaas and Craik 2015). Standardization would enable a seamless exchange of data, fostering collaboration and driving innovation.

Many current bioinformatic techniques used to generate genomic, transcriptomic, and proteomic datasets for venomous organisms may have relied on methods that have since been improved or changed or that are nonstandard. These limitations stem partly from the fact that many venomous organisms are often categorized as “nonmodel organisms” by the broader scientific community, which has historically prioritized the development of advanced ‘omics approaches for model species (Armengaud et al. 2014; Muth et al. 2018; Calvete et al. 2021). This has led to a significant gap in the tools and resources available for studying the unique and often complex biology of venomous taxa that VenomsBase (Fig. 2) would address. Visualization tools built into VenomsBase accompanying genome browsers and protein structure renderings would enable intuitive access to complex datasets, making it easier for interdisciplinary researchers to interact with these diverse data types. These tools could foster collaboration between traditionally distinct fields.

VenomsBase will transform venom research by centralizing and standardizing data, bridging disciplines, and enhancing interdisciplinary collaboration. By unifying fragmented datasets and enabling comparative analyses, it will advance fundamental research while unlocking applications in pharmaceuticals, biocontrol, and cosmetics. This integrated platform exemplifies the power of centralized infrastructure to drive scientific innovation.

## VenomsBase: infrastructure that both serves and integrates research communities

VenomsBase will serve as a critical infrastructure for data integration, collaboration, and innovation, providing an equitable platform for researchers across disciplines and geographic regions (Fig. 2). By enabling the sharing of protocols and methodologies, VenomsBase will help standardize best practices in venom research while supporting global accessibility. Through its open-access design and commitment to FAIR principles, it will foster cross-disciplinary knowledge exchange. It will ensure researchers have access to cutting-edge techniques, such as transcriptomic workflows, venom protein isolation methods, and bioinformatic analysis pipelines. Experts could contribute specialized protocols, which others could adopt or adapt. It can incorporate existing cloud platforms such as Cyverse (Swetnam et al. 2024) with integrated GitHub repositories and UCSC genome browser (Lee et al. 2020) for new organisms, environments, or experimental conditions, promoting inclusivity and innovation. Additionally, the database could provide training resources, facilitate knowledge exchange, and create opportunities for researchers from underrepresented regions to participate in collaborative projects.

Venoms hold untapped potential for addressing global health challenges, including envenomation, which affects over 5 million people annually. Snakebites alone cause significant morbidity and mortality, particularly in under-resourced regions (Simpson 2008; Williams et al. 2011; Patikorn et al. 2022). By developing centralized, open-access databases and fostering collaborative networks, researchers in these regions could gain access to critical venom protein sequencing data, genomic datasets, and bioinformatic tools without incurring prohibitive costs. Additionally, investing in local venoms research capacity, through partnerships with universities, hospitals, and public health organizations, could lead to improved diagnostics and antivenom development tailored to regional snake, spider, or scorpion species (Fry et al. 2003; Yu et al. 2020). Addressing the global disparities in venom research by providing cost-effective and scalable solutions would reduce the burden of snakebite envenomation and also empower researchers in low-resource settings to contribute meaningfully to scientific discoveries in toxinology, evolutionary biology, and pharmacology.

Online educational infrastructure would also help bridge the research gap among educational and research institutions that would benefit from applying these resources. An online infrastructure hosting standardized high-quality venom resources, along with tutorials and instructional resources, would provide hands-on data analysis training and introductory opportunities for functional genomics (Brown 2016; Gao and Guo 2023). This infrastructure could bridge the gap between basic research and translational applications, accelerating the development of antivenoms and novel drugs.

In conclusion, by outlining best practices toward collaborative venom-based research, VenomsBase represents a transformative leap in venoms research (Fig. 2). A centralized platform like VenomsBase would pave the way for groundbreaking discoveries in evolutionary biology, pharmacology, and ecological science by addressing the limitations of fragmented datasets, standardizing nomenclature, data collection, and integrating multidisciplinary and multimodal datasets. VenomsBase exemplifies the power of centralized, interdisciplinary infrastructure to drive scientific innovation by unifying resources, increasing comparative studies and facilitating flexibility in data analyses.

## References

[bib1] Afgan E, Baker D, Batut B, van den Beek M, Bouvier D, Čech M, Chilton J, Clements D, Coraor N, Grüning BA et al. 2018. The Galaxy platform for accessible, reproducible and collaborative biomedical analyses: 2018 update. Nucleic Acids Res 46:W537–44. 29790989 10.1093/nar/gky379 PMC6030816

[bib2] Afroz A, Siddiquea BN, Chowdhury HA, Jackson TN, Watt AD. 2024. Snakebite envenoming: a systematic review and meta-analysis of global morbidity and mortality. PLoS Negl Trop Dis 18:e0012080. 38574167 10.1371/journal.pntd.0012080 PMC11020954

[bib3] Ahyong S, Boyko CB, Bernot J, Brandão SN, Daly M, De Grave S, de Voogd NJ, Gofas S, Hernandez F, Hughes L et al. 2025. World Register of Marine Species. https://www.marinespecies.org at VLIZ. Accessed 2025-05-23. 10.14284/170

[bib4] Arbuckle K . 2020. From molecules to macroevolution: venom as a model system for evolutionary biology across levels of life. Toxicon: X 6:100034. 32550589 10.1016/j.toxcx.2020.100034 PMC7285901

[bib5] Armengaud J, Trapp J, Pible O, Geffard O, Chaumot A, Hartmann EM. 2014. Non-model organisms, a species endangered by proteogenomics. J Proteom Spec Issue Proteom Non-model Org 105:5–18. 10.1016/j.jprot.2014.01.007 24440519

[bib6] Arshinoff BI, Cary GA, Karimi K, Foley S, Agalakov S, Delgado F, Lotay VS, Ku CJ, Pells TJ, Beatman TR et al. 2022. Echinobase: leveraging an extant model organism database to build a knowledgebase supporting research on the genomics and biology of echinoderms. Nucleic Acids Res 50:D970–9. 34791383 10.1093/nar/gkab1005 PMC8728261

[bib7] Attwood TK, Blackford S, Brazas MD, Davies A, Schneider MV. 2019. A global perspective on evolving bioinformatics and data science training needs. Briefings Bioinf 20:398–404. 10.1093/bib/bbx100 PMC6433731 28968751

[bib8] Auwera Gvd, O'Connor BD. 2020. Genomics in the cloud: using Docker, GATK, and WDL in Terra O'Reilly Media, Incorporated. https://catalog.nlm.nih.gov/discovery/fulldisplay/alma9917773213406676/1445500

[bib9] Avella I, Wüster W, Luiselli L, Martínez-Freiría F. 2022. Toxic habits: an analysis of general trends and biases in snake venom research. Toxins 14:884. 36548781 10.3390/toxins14120884 PMC9783912

[bib10] Barua A, Mikheyev AS. 2021. An ancient, conserved gene regulatory network led to the rise of oral venom systems. Proc Natl Acad Sci USA 118:e2021311118. 33782124 10.1073/pnas.2021311118 PMC8040605

[bib11] Batko K, Ślęzak A. 2022. The use of Big Data Analytics in healthcare. J Big Data 9:1–24. 10.1186/s40537-021-00553-4 PMC8733917 35013701

[bib12] Berardini TZ, Reiser L, Li D, Mezheritsky Y, Muller R, Strait E, Huala E. 2015. The arabidopsis information resource: making and mining the “gold standard” annotated reference plant genome. Genesis 53:474–85. 26201819 10.1002/dvg.22877 PMC4545719

[bib13] Brazas MD, Ouellette BFF. 2013. Navigating the changing learning landscape: perspective from bioinformatics.Ca. Briefings Bioinf 14:556–62. 10.1093/bib/bbt016 PMC3771234 23515468

[bib14] Breitwieser L, Hesam A, de Montigny J, Vavourakis V, Iosif A, Jennings J, Kaiser M, Manca M, Di Meglio A, Al-Ars Z et al. 2022. BioDynaMo: a modular platform for high-performance agent-based simulation. Bioinformatics 38:453–60. 34529036 10.1093/bioinformatics/btab649 PMC8723141

[bib15] Brown JAL . 2016. Evaluating the effectiveness of a practical inquiry-based learning bioinformatics module on undergraduate student engagement and applied skills. Biochem Mol Bio Educ 44:304–13. 27161812 10.1002/bmb.20954

[bib16] Calvete JJ, Lomonte B, Saviola AJ, Bonilla F, Sasa M, Williams DJ, Undheim EAB, Sunagar K, Jackson TNW. 2021. Mutual enlightenment: a toolbox of concepts and methods for integrating evolutionary and clinical toxinology via snake venomics and the contextual stance. Toxicon X: 9-10:100070. 34195606 10.1016/j.toxcx.2021.100070 PMC8234350

[bib17] Calvete JJ, Lomonte B, Saviola AJ, Calderón Celis F, Ruiz Encinar J. 2024. Quantification of snake venom proteomes by mass spectrometry-considerations and perspectives. Mass Spectrom Rev 43:977–97. 37155340 10.1002/mas.21850

[bib18] Casewell NR, Wüster W, Vonk FJ, Harrison RA, Fry BG. 2013. Complex cocktails: the evolutionary novelty of venoms. Trends Ecol Evol 28:219–29. 23219381 10.1016/j.tree.2012.10.020

[bib19] Choteau SA, Wagner A, Pierre P, Spinelli L, Brun C. 2021. MetamORF: a repository of unique short open reading frames identified by both experimental and computational approaches for gene and metagene analyses. Database 2021:baab032. 34156446 10.1093/database/baab032 PMC8218702

[bib20] Christou GA, Katsiki N, Blundell J, Fruhbeck G, Kiortsis DN. 2019. Semaglutide as a promising antiobesity drug. Obes Rev 20:805–15. 30768766 10.1111/obr.12839

[bib21] Clark K, Karsch-Mizrachi I, Lipman DJ, Ostell J, Sayers EW. 2016. GenBank. Nucleic Acids Res 44:D67–72. 26590407 10.1093/nar/gkv1276 PMC4702903

[bib22] Coelho LP, Santos-Júnior CD, de la Fuente-Nunez C. 2024. Challenges in computational discovery of bioactive peptides in ’omics data. Proteomics 24:2300105. 10.1002/pmic.202300105 PMC11537280 38458994

[bib23] de Oliveira AN, Soares AM, Da Silva SL. 2023. Why to study peptides from venomous and poisonous animals? Int J Pept Res Ther 29:76.

[bib24] DiFrisco J, Jaeger J. 2020. Genetic causation in complex regulatory systems: an integrative dynamic perspective. Bioessays 42:1900226. 10.1002/bies.201900226 32449193

[bib25] Di Muri C, Pulieri M, Raho D, Muresan AN, Tarallo A, Titocci J, Nestola E, Basset A, Mazzoni S, Rosati I. 2024. Assessing semantic interoperability in environmental sciences: variety of approaches and semantic artefacts. Sci Data 11:1055. 39333503 10.1038/s41597-024-03669-3 PMC11437166

[bib26] Dresler J, Avella I, Damm M, Dersch L, Krämer J, Vilcinskas A, Lüddecke T. 2024. A roadmap to the enzymes from spider venom: biochemical ecology, molecular diversity, and value for the bioeconomy. Front Arachn Sci 3:1445500.

[bib27] Dyer SC, Austine-Orimoloye O, Azov AG, Barba M, Barnes I, Vianey Barrera-Enriquez P, Becker A, Bennett R, Beracochea M, Berry A et al. 2025. Ensembl 2025, Nucleic Acids Res 53: D948–57. 39656687 10.1093/nar/gkae1071 PMC11701638

[bib28] Emms DM, Kelly S. 2019. OrthoFinder: phylogenetic orthology inference for comparative genomics. Genome Biol 20:238. 31727128 10.1186/s13059-019-1832-y PMC6857279

[bib29] European Commission: Directorate-General for Research and Innovation . 2018. Turning FAIR Into Reality: Final Report and Action Plan from the European Commission Expert Group on FAIR Data Publications Office.

[bib30] Frisvold GB, Moss SM, Hodgson A, Maxon ME. 2021. Understanding the U.S. Bioeconomy: a new definition and landscape. Sustainability 13:1627.

[bib31] Fry BG, Winkel KD, Wickramaratna JC, Hodgson WC, Wüster W. 2003. Effectiveness of snake antivenom: species and regional venom variation and its clinical impact. J Toxicol Toxin Rev 22:23–34.

[bib32] Gao L, Guo M. 2023. A course-based undergraduate research experience for bioinformatics education in undergraduate students. Biochem Molecular Bio Educ 51:189–99. 10.1002/bmb.21710 36779350

[bib33] GBIF.org . 2025. GBIF Home Page. https://www.gbif.org/

[bib34] Geneviève LD, Ray N, Chappuis F, Alcoba G, Mondardini MR, Bolon I, Castañeda RRD. 2018. Participatory approaches and open data on venomous snakes: a neglected opportunity in the global snakebite crisis?. PLoS Neglected Tropic Dis, 12, e0006162. 10.1371/journal.pntd.0006162 PMC5843214 29518075

[bib35] Gopalan SS, Perry BW, Schield DR, Smith CF, Mackessy SP, Castoe TA. 2022. Origins, genomic structure and copy number variation of snake venom myotoxins. Toxicon 216:92–106. 35820472 10.1016/j.toxicon.2022.06.014

[bib36] Hargreaves AD, Mulley JF. 2014. A plea for standardized nomenclature of snake venom toxins. Toxicon 90:351–3. 25193251 10.1016/j.toxicon.2014.08.070

[bib37] Holford M, Daly M, King GF, Norton RS. 2018. Venoms to the rescue. Science 361:842–4. 30166472 10.1126/science.aau7761

[bib38] Holmes DE . 2018. Big Data: a Very Short Introduction, Very Short Introductions Oxford, New York: Oxford University Press.

[bib39] i5K Consortium . 2013. The i5K Initiative: advancing arthropod genomics for knowledge, Human health, agriculture, and the environment. J Hered 104:595–600. 23940263 10.1093/jhered/est050 PMC4046820

[bib40] Jungo F, Bougueleret L, Xenarios I, Poux S. 2012. The UniProtKB/Swiss-Prot Tox-Prot program: a central hub of integrated venom protein data. Toxicon Adv Basic Translat Venomics 60:551–7. 10.1016/j.toxicon.2012.03.010 PMC3393831 22465017

[bib41] Jungo F, Estreicher A, Bairoch A, Bougueleret L, Xenarios I. 2010. Animal toxins: how is complexity represented in databases? Toxins 2:262–82. 22069583 10.3390/toxins2020262 PMC3202812

[bib42] Kaas Q, Craik DJ. 2015. Bioinformatics-aided venomics. Toxins 7:2159–87. 26110505 10.3390/toxins7062159 PMC4488696

[bib43] Kaas Q, Yu R, Jin A-H, Dutertre S, Craik DJ. 2012. ConoServer: updated content, knowledge, and discovery tools in the conopeptide database. Nucleic Acids Res 40:D325. 22058133 10.1093/nar/gkr886 PMC3245185

[bib44] Kasturiratne A, Wickremasinghe AR, Silva Nd, Gunawardena NK, Pathmeswaran A, Premaratna R, Savioli L, Lalloo DG, Silva HJd. 2008. The global burden of snakebite: a literature analysis and modelling based on regional estimates of envenoming and deaths. PLoS Med 5:e218. 18986210 10.1371/journal.pmed.0050218 PMC2577696

[bib45] King G . 2015. Venoms to Drugs: Venom as a Source for the Development of Human Therapeutics. Cambridge, UK: Royal Society of Chemistry.

[bib46] Kini RM . 2020. Toxinology provides multidirectional and multidimensional opportunities: a personal perspective. Toxicon X 6:100039. 32550594 10.1016/j.toxcx.2020.100039 PMC7285919

[bib47] Kuzmenkov AI, Krylov NA, Chugunov AO, Grishin EV, Vassilevski AA. 2016. Kalium: a database of potassium channel toxins from scorpion venom. Database 2016:baw056. 27087309 10.1093/database/baw056 PMC4834203

[bib48] Lee CM, Barber GP, Casper J, Clawson H, Diekhans M, Gonzalez JN, Hinrichs AS, Lee BT, Nassar LR, Powell CC et al. 2020. UCSC Genome Browser enters 20th year. Nucleic Acids Res 48:D756–61. 31691824 10.1093/nar/gkz1012 PMC7145642

[bib49] Lehne M, Sass J, Essenwanger A, Schepers J, Thun S. 2019. Why digital medicine depends on interoperability. NPJ Digit Med 2:1–5. 31453374 10.1038/s41746-019-0158-1 PMC6702215

[bib50] Lowman M, D'Avanzo C, Brewer C. 2009. A National Ecological Network for Research and Education. Science 323:1172–3. 19251614 10.1126/science.1166945

[bib51] Martinson EO, Mrinalini, Kelkar YD, Chang C-H, Werren JH. 2017. The evolution of venom by Co-option of single-copy genes. Curr Biol 27:2007–2013.e8.e8. 28648823 10.1016/j.cub.2017.05.032 PMC5719492

[bib52] McLaren W, Gil L, Hunt SE, Riat HS, Ritchie GRS, Thormann A, Flicek P, Cunningham F. 2016. The Ensembl variant effect predictor. Genome Biol 17:122. 27268795 10.1186/s13059-016-0974-4 PMC4893825

[bib53] Muth T, Hartkopf F, Vaudel M, Renard BY. 2018. A potential golden age to come—current tools, recent use cases, and future avenues for De Novo sequencing in proteomics. Proteomics 18:1700150. 10.1002/pmic.201700150 29968278

[bib54] Muttenthaler M, King GF, Adams DJ, Alewood PF. 2021. Trends in peptide drug discovery. Nat Rev Drug Discov 20:309–25. 33536635 10.1038/s41573-020-00135-8

[bib55] Nachtigall PG, Durham AM, Rokyta DR, Junqueira-de-Azevedo ILM. 2024. ToxCodAn-genome: an automated pipeline for toxin-gene annotation in genome assembly of venomous lineages. GigaScience 13:giad116. 38241143 10.1093/gigascience/giad116 PMC10797961

[bib56] Nachtigall PG, Rautsaw RM, Ellsworth SA, Mason AJ, Rokyta DR, Parkinson CL, Junqueira-de-Azevedo ILM. 2021. ToxCodAn: a new toxin annotator and guide to venom gland transcriptomics. Briefings Bioinf 22:bbab095. 10.1093/bib/bbab095 33866357

[bib57] Ogawa T, Oda-Ueda N, Hisata K, Nakamura H, Chijiwa T, Hattori S, Isomoto A, Yugeta H, Yamasaki S, Fukumaki Y et al. 2019. Alternative mRNA splicing in three venom families underlying a possible production of divergent venom proteins of the Habu snake, protobothrops flavoviridis. Toxins 11:581. 31600994 10.3390/toxins11100581 PMC6832531

[bib58] Oliveira JS, Fuentes-Silva D, King GF. 2012. Development of a rational nomenclature for naming peptide and protein toxins from sea anemones. Toxicon Adv Basic Translat Venomics 60:539–50. 10.1016/j.toxicon.2012.05.020 22683676

[bib59] Öztürk-Çolak A, Marygold SJ, Antonazzo G, Attrill H, Goutte-Gattat D, Jenkins VK, Matthews BB, Millburn G, dos Santos G, Tabone CJ, FlyBase Consortium. 2024. FlyBase: updates to the Drosophila genes and genomes database. Genetics 227:iyad211. 38301657 10.1093/genetics/iyad211 PMC11075543

[bib60] Palmer JM, Stajich J. 2020. Funannotate v1.8.1: eukaryotic genome annotation (v1.8). Zenodo 10.5281/zenodo.1134477

[bib61] Patikorn C, Ismail AK, Abidin SAZ, Blanco FB, Blessmann J, Choumlivong K, Comandante JD, Doan UV, Ismail ZM, Khine YY et al. 2022. Situation of snakebite, antivenom market and access to antivenoms in ASEAN countries. BMJ Glob Health 7: e007639. 10.1136/bmjgh-2021-007639 PMC8928241 35296460

[bib62] Perry BW, Gopalan SS, Pasquesi GI, Schield DR, Westfall AK, Smith CF, Koludarov I, Chippindale PT, Pellegrino MW, Chuong EB. 2022. Snake venom gene expression is coordinated by novel regulatory architecture and the integration of multiple co-opted vertebrate pathways. Genome Res 32: 1058–73. 35649579 10.1101/gr.276251.121 PMC9248877

[bib63] Pineda SS, Chaumeil P-A, Kunert A, Kaas Q, Thang MWC, Le L, Nuhn M, Herzig V, Saez NJ, Cristofori-Armstrong B et al. 2018. ArachnoServer 3.0: an online resource for automated discovery, analysis and annotation of spider toxins. Bioinformatics 34:1074–6. 29069336 10.1093/bioinformatics/btx661

[bib64] Politano G, Di Carlo S, Benso A. 2019. “One DB to rule them all.”—The RING: a regulatory INteraction graph combining TFs, genes/proteins, SNPs, diseases and drugs. Database 2019:baz108. 31682269 10.1093/database/baz108 PMC6827393

[bib65] Prentis PJ, Pavasovic A, Norton RS. 2018. Sea Anemones: quiet achievers in the field of peptide toxins. Toxins 10:36. 29316700 10.3390/toxins10010036 PMC5793123

[bib66] Puzari U, Das B, Mukherjee AK. 2025. Advancements in diagnostic techniques for scorpion venom identification: a comprehensive review. Toxicon 253:108191. 39613267 10.1016/j.toxicon.2024.108191

[bib67] Rahrooh A, Garlid AO, Bartlett K, Coons W, Petousis P, Hsu W, Bui AAT. 2024. Towards a framework for interoperability and reproducibility of predictive models. J Biomed Inform 149:104551. 38000765 10.1016/j.jbi.2023.104551

[bib68] Romano JD, Tatonetti NP. 2015. VenomKB, a new knowledge base for facilitating the validation of putative venom therapies. Sci Data 2:150065. 26601758 10.1038/sdata.2015.65 PMC4658572

[bib69] Schendel V, Rash LD, Jenner RA, Undheim EAB. 2019. The diversity of venom: the importance of behavior and venom system morphology in understanding its ecology and evolution. Toxins 11:666. 31739590 10.3390/toxins11110666 PMC6891279

[bib70] Schield DR, Perry BW, Adams RH, Holding ML, Nikolakis ZL, Gopalan SS, Smith CF, Parker JM, Meik JM, DeGiorgio M et al. 2022. The roles of balancing selection and recombination in the evolution of rattlesnake venom. Nat Ecol Evol 6:1367–80. 35851850 10.1038/s41559-022-01829-5 PMC9888523

[bib71] Shaffer CD, Alvarez C, Bailey C, Barnard D, Bhalla S, Chandrasekaran C, Chandrasekaran V, Chung H-M, Dorer DR, Du C et al. 2010. The Genomics Education Partnership: successful integration of research into laboratory classes at a diverse group of undergraduate institutions. CBE Life Sci Educ 9:55–69. 20194808 10.1187/09-11-0087 PMC2830162

[bib72] Sima AC, Mendes de Farias T, Zbinden E, Anisimova M, Gil M, Stockinger H, Stockinger K, Robinson-Rechavi M, Dessimoz C. 2019. Enabling semantic queries across federated bioinformatics databases. Database 2019:baz106. 31697362 10.1093/database/baz106 PMC6836710

[bib73] Simpson ID . 2008. Time for an alternative perspective: the eternal problem of supply and quality of anti snake venom in the developing world—“it's the economy, stupid”. Wilderness Environ Med 19:186–94. 18715131 10.1580/08-WEME-CON-194.1

[bib74] Smallwood TB, Clark RJ. 2021. Advances in venom peptide drug discovery: where are we at and where are we heading? Expert Opin Drug Discovery 16:1163–73. 10.1080/17460441.2021.1922386 33914674

[bib75] Smith EG, Surm JM, Macrander J, Simhi A, Amir G, Sachkova MY, Lewandowska M, Reitzel AM, Moran Y. 2023. Micro and macroevolution of sea anemone venom phenotype. Nat Commun 14:249. 36646703 10.1038/s41467-023-35794-9 PMC9842752

[bib76] Sternberg PW, Van Auken K, Wang Q, Wright A, Yook K, Zarowiecki M, Arnaboldi V, Becerra A, Brown S, Cain S et al. 2024. WormBase 2024: status and transitioning to Alliance infrastructure. Genetics 227:iyae050. 38573366 10.1093/genetics/iyae050 PMC11075546

[bib77] Sunagar K, Morgenstern D, Reitzel AM, Moran Y. 2016. Ecological venomics: how genomics, transcriptomics and proteomics can shed new light on the ecology and evolution of venom. J Proteom Proteom Evol Ecol 135:62–72. 10.1016/j.jprot.2015.09.015 26385003

[bib78] Swetnam TL, Antin PB, Bartelme R, Bucksch A, Camhy D, Chism G et al. (2024). CyVerse: Cyberinfrastructure for open science. PLoS Comput Biol 20:e1011270. 10.1371/journal.pcbi.1011270 PMC10878509 38324613

[bib79] The UniProt Consortium . 2025. UniProt: the Universal protein knowledgebase in 2025. Nucleic Acids Res 53:D609–17. 39552041 10.1093/nar/gkae1010 PMC11701636

[bib80] Treangen TJ, Koren S, Sommer DD, Liu B, Astrovskaya I, Ondov B, Darling AE, Phillippy AM, Pop M. 2013. MetAMOS: a modular and open source metagenomic assembly and analysis pipeline. Genome Biol 14:R2. 23320958 10.1186/gb-2013-14-1-r2 PMC4053804

[bib81] Vonk FJ, Casewell NR, Henkel CV, Heimberg AM, Jansen HJ, McCleary RJ, Kerkkamp HM, Vos RA, Guerreiro I, Calvete JJ. 2013. The king cobra genome reveals dynamic gene evolution and adaptation in the snake venom system. Proc Natl Acad Sci USA 110:20651–6. 24297900 10.1073/pnas.1314702110 PMC3870661

[bib82] von Reumont BM, Anderluh G, Antunes A, Ayvazyan N, Beis D, Caliskan F, Crnković A, Damm M, Dutertre S, Ellgaard L et al. 2022. Modern venomics—current insights, novel methods, and future perspectives in biological and applied animal venom research. GigaScience 11:giac048. 35640874 10.1093/gigascience/giac048 PMC9155608

[bib83] Wilkinson MD, Dumontier M, Aalbersberg IJ, Appleton G, Axton M, Baak A, Blomberg N, Boiten J-W, da Silva Santos LB, Bourne PE et al. 2016. The FAIR Guiding Principles for scientific data management and stewardship. Sci Data 3:160018. 26978244 10.1038/sdata.2016.18 PMC4792175

[bib84] Wilkinson SR, Aloqalaa M, Belhajjame K et al. 2025. Applying the FAIR Principles to computational workflows. Sci Data 12:328. 10.1038/s41597-025-04451-9 PMC11850811 39994238

[bib85] Williams DJ, Gutiérrez J-M, Calvete JJ, Wüster W, Ratanabanangkoon K, Paiva O, Brown NI, Casewell NR, Harrison RA, Rowley PD et al. 2011. Ending the drought: new strategies for improving the flow of affordable, effective antivenoms in Asia and Africa. J Proteom “Omic” Stud Neglected Tropic Dis 74:1735–67. 10.1016/j.jprot.2011.05.027 21640209

[bib86] Wong ESW, Belov K. 2012. Venom evolution through gene duplications. Gene 496:1–7. 22285376 10.1016/j.gene.2012.01.009

[bib87] Ye X, He C, Yang Y, Sun YH, Xiong S, Chan KC, Si Y, Xiao S, Zhao X, Lin H et al. 2023. Comprehensive isoform-level analysis reveals the contribution of alternative isoforms to venom evolution and repertoire diversity. Genome Res 33:1554–67. 37798117 10.1101/gr.277707.123 PMC10620052

[bib88] Yu C, Yu H, Li P. 2020. Highlights of animal venom research on the geographical variations of toxin components, toxicities and envenomation therapy. Int J Biol Macromol 165:2994–3006. 33122066 10.1016/j.ijbiomac.2020.10.190

[bib89] Zancolli G, Casewell NR. 2020. Venom systems as models for studying the origin and regulation of evolutionary novelties. Mol Biol Evol 37:2777–90. 32462210 10.1093/molbev/msaa133

[bib90] Zancolli G, von Reumont BM, Anderluh G, Caliskan F, Chiusano ML, Fröhlich J, Hapeshi E, Hempel B-F, Ikonomopoulou MP, Jungo F et al. 2024. Web of venom: exploration of big data resources in animal toxin research. GigaScience 13:giae054. 39250076 10.1093/gigascience/giae054 PMC11382406

[bib91] Zelanis A, Keiji Tashima A. 2014. Unraveling snake venom complexity with ‘omics’ approaches: challenges and perspectives. Toxicon 87:131–4. 24878375 10.1016/j.toxicon.2014.05.011

